# 1875. Factors associated with unfavorable tuberculosis treatment outcomes, Almaty, Kazakhstan, 2018-2021

**DOI:** 10.1093/ofid/ofad500.1703

**Published:** 2023-11-27

**Authors:** Malika Gabdullina, Saya Gazezova, Gulzhan Ayapova, Manar Smagul, Roberta Horth, Dilyara Nabirova

**Affiliations:** Central Asia Field Epidemiology Training Program, Almaty, Almaty, Kazakhstan; Central Asia Field Epidemiology Training Program, Almaty, Almaty, Kazakhstan; Central Asia Field Epidemiology Training Program, Almaty, Almaty, Kazakhstan; Scientific and practical center of sanitary-epidemiological examination and monitoring, branch of the National Center for Public Health, Almaty, Kazakhstan, Almaty, Almaty, Kazakhstan; US Centers for Disease Control and Prevention, Dulles, Virginia; CDC Central Asia office, Almaty, Almaty, Kazakhstan

## Abstract

**Background:**

The COVID-19 pandemic negatively influenced the availability of TB related services around the world, including detection, diagnosis and treatment. The purpose of this research is to assess the impact of the COVID-19 pandemic on TB treatment outcomes in Almaty, and to determine risk factors associated with said outcomes by examining data from before (2018-2019) and during the pandemic (2020-2021).

**Methods:**

We conducted a retrospective cohort study among all people newly diagnosed with drug-sensitive tuberculosis over 18 years old who initiated treatment from 2018-2021 in Almaty. We abstracted data from the national tuberculosis electronic database. Unfavorable treatment outcomes were sorted as follows: ineffective treatment, death, loss for follow-up, results not evaluated, and transferred for treatment with second-line anti-TB drugs. We used multivariate Poisson regression to calculate adjusted relative risk [aRR] and 95% confidence intervals [95%CI].

Risk factors associated with TB treatment outcomes among people newly diagnosed with drug-susceptible TB, Almaty, Kazakhstan, 2018-2022

**Figure:**
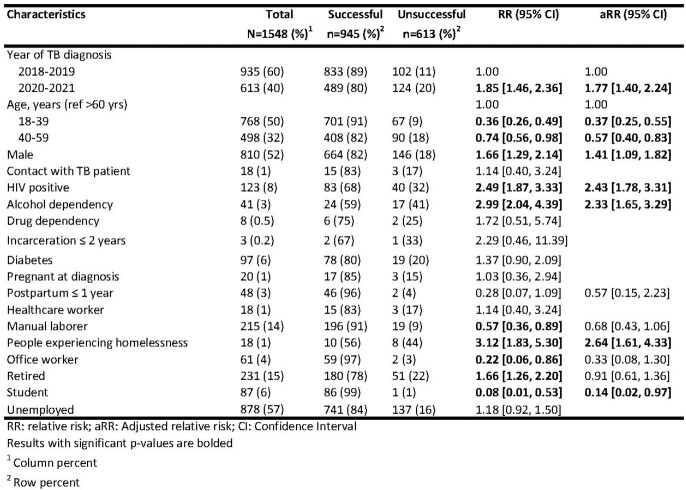

**Results:**

Among 1548 people newly diagnosed with TB during the study period, average age of was 43 years (range 18-93) and 52% were male. A higher proportion of people had unfavorable outcomes during the pandemic (2020-21) than before the pandemic (2018-19) (20% vs 11%, respectively, and aRR=1.8; CI: 1.4-2.2). Among those with unsuccessful outcomes, 1% were lost to follow-up, 3% had treatment failures, and 9% died. Risk factors for unfavorable TB treatment outcomes were: being male (aRR=1.4, 95%CI=1.1-1.8), having HIV (aRR=2.4, 95%CI=1.8-3.3), having drinking disorder (aRR=2.3, 95%CI=1.6-3.3) and experiencing homelessness (aRR=2.6, 95%CI=1.5-4.3) (Table 1). Protective factors for unfavorable TB treatment outcomes were: being 18-39 years old (aRR=0.4, 95%CI=0.3-0.6), 40-59 years old (aRR=0.6, 95%CI=0.4-0.8), and being student (aRR=0.1, 95%CI=0.02-0.9).

**Conclusion:**

COVID-19 pandemic was associated with unfavorable treatment outcomes for people newly diagnosed with drug-sensitive tuberculosis in Almaty, Kazakhstan. People with lower social stability were at increased risk. Results point to the need for improved continuity of care for TB treatment for people at increased risk of unfavorable outcomes, especially during disease outbreaks and pandemics.

**Disclosures:**

**All Authors**: No reported disclosures

